# Direct and Indirect Effects of Autism Spectrum Disorder Severity on Dental Health Status in Children and Adolescents: A Structural Equation Modeling Approach

**DOI:** 10.3390/medicina62010086

**Published:** 2025-12-31

**Authors:** Eglė Slabšinskienė, Lukrecija Lazarukaitė, Nikolajus Kurenkovas, Aistė Kavaliauskienė, Rūta Grigalauskienė, Ingrida Vasiliauskienė, Apolinaras Zaborskis

**Affiliations:** 1 Department of Preventive and Paediatric Dentistry, Faculty of Odontology, Medical Academy, Lithuanian University of Health Sciences, J. Lukšos-Daumanto st. 6, LT-50106 Kaunas, Lithuania; 2 Faculty of Medicine, Medical Academy, Lithuanian University of Health Sciences, Mickevičiaus st. 9, LT-44307 Kaunas, Lithuania; 3 Department of Orthodontics, Faculty of Odontology, Medical Academy, Lithuanian University of Health Sciences, J. Lukšos-Daumanto st. 6, LT-50106 Kaunas, Lithuania; 4 Department of Preventive Medicine & Health Research Institute, Faculty of Public Health, Medical Academy, Mickevičiaus st. 9, LT-44307 Kaunas, Lithuania

**Keywords:** autism spectrum disorders, dental health, toothbrushing, diet, mediating effect, path model

## Abstract

*Background and Objectives*: Current evidence remains insufficient to determine whether the impact of autism spectrum disorder (ASD) on dental health is primarily mediated through oral hygiene and dietary habits or through direct effects of the disorder itself. This study examined the theoretical pathways through which ASD severity and toothbrushing-related and dietary-choice-related factors influence dental health in autistic children and adolescents. *Materials and Methods*: A cross-sectional study was conducted with 399 mothers reporting on their autistic children (aged 2–18 years, mean = 7.8). The exclusion criterion was being older than 18 years. Data included parent-reported data about ASD severity, dental health status, willingness to brush teeth, and dietary quality (assessed using the Diet Quality Inventory). Structural Equation Modeling (SEM) was used to analyze the direct and indirect effects of ASD severity on dental health, with probit regression coefficients estimated using the WLSMV method. *Results*: Parent-reported variables of ASD severity, diet quality, and toothbrushing willingness together explained 37% of the variance in dental health. The direct effect of ASD severity on dental health was 0.199 (*p* = 0.039). The indirect effect via toothbrushing was 0.137 (*p* = 0.006), and via diet quality, it was 0.070 (*p* = 0.020). The total indirect effect of ASD on dental health was 0.207 (*p* = 0.026), which was approximately as strong as the direct effect. The associations among the studied variables were statistically equivalent across sex and age groups. *Conclusions*: Parent-reported ASD severity shows significant association with dental health outcomes, both directly and indirectly, with toothbrushing behavior emerging as the primary mediator. Interventions that promote regular brushing (and, to a lesser extent, healthier eating) may help to reduce the dental health disparities associated with autism.

## 1. Introduction

The prevalence of autism spectrum disorder (ASD) has risen in recent years. As stated by the World Health Organization, about 1 in 127 children worldwide has ASD [1]. In 2023, the Centers for Disease Control and Prevention (CDC) reported that approximately 1 in 31 (or 3.226%) children have this condition in the United States [2]. Similar data was reported by the Lithuanian Institute of Hygiene, which showed that the number of children with autism per 100,000 increased from 105.6 in 2016 to 238.9 in 2021 in Lithuania [3]. Children with ASD are at a higher risk for a variety of health problems, including dental issues, which result from both direct biological and indirect behavioral factors related to the disorder [4,5,6].

It is evident that oral health plays a crucial role in overall health and quality of life, especially in children, amongst whom dental caries is the most prevalent condition that requires dental care [7]. A prospective longitudinal study conducted in 2021 reported that parents of children and adolescents with ASD considered that dental treatment improved their child’s oral health-related quality of life [8]. According to prior research, children with ASD may be more susceptible to dental caries and other oral health issues, and they have a greater vulnerability to functional and psychosocial difficulties linked to poor dental health as well [9,10]. Because of communication difficulties, sensory sensitivity, and behavioral challenges they experience, children with ASD frequently have trouble accessing dental care, which makes maintaining good oral hygiene and going to the dentist especially difficult [11,12]. According to the recent literature, children with ASD are more likely than their typically developing peers to suffer from dental caries and periodontal disease, mainly due to dietary habits and challenges in maintaining oral hygiene routines [11,13].

ASD-related behavioral factors, such as sensory sensitivities or cognitive limitations, can make it difficult to develop good brushing habits, which eventually leads to poor oral hygiene. It was found that children with ASD used toothpaste and brushed their teeth less frequently and depended more on parental help [14].

Evaluating dietary factors in children with ASD is important for understanding their dental health status. Many children with ASD have specific food preferences and often avoid healthier choices, selecting foods high in sugar and refined carbohydrates due to sensory aversion [15,16]. This preference can greatly increase their risk for dental caries.

Regarding other indirect influencing factors, there is a lack of studies on the impact of parental factors on the oral health of children with ASD; however, parental attitudes and practices regarding oral health are also influential [17]. Parental participation is essential in order to maintain good oral health in children with ASD. Parental satisfaction is one of the most influential factors for ensuring good dental status in children [18]. Therefore, negative parental attitudes are linked to poorer dental health in children with ASD [19]. As a result, the indirect effects of autism on dental health are varied and include daily care, lifestyle challenges and environmental barriers that together lead to worse outcomes for these children [20,21,22].

In conclusion, autistic children (and the same applies to adolescents) tend to have poorer oral health and a higher prevalence of dental caries in comparison to their typically developing peers. Poor dietary and oral hygiene habits, parental stress, and low socioeconomic status all contribute to this outcome. However, there is a lack of knowledge in the current literature regarding the role and importance of these factors in the pathway between ASD and oral health. Few studies have separated factors that act via direct versus indirect pathways. For example, some articles focus on behavioral and social characteristics, while others identify structural barriers to dental care as indirect factors that affect oral health outcomes [20,22]. Finally, to our knowledge, no studies have addressed the fundamental question of how dental hygiene behaviors and dietary choices compare in importance to the effects of autism itself for the oral health of children with ASD.

To address this gap, this study aimed to investigate the pathways through which ASD severity influences dental health in autistic children and adolescents. We hypothesized that the severity of ASD is associated with dental health not only directly but also indirectly, and with equal importance, through toothbrushing habits and dietary choices. To test this, we applied a simple path model to distinguish between the direct and indirect pathways within a Structural Equation Modeling (SEM) framework [23,24].

## 2. Materials and Methods

### 2.1. Study Population and Recruitment

This study was approved by the Advisory Board of the Bioethics Centre at the Lithuanian University of Health Sciences (License No. 2024-BEC2-1063, issued on 12 November 2024). The sample size for this study was estimated based on the assumption that data analysis would be conducted using Structural Equation Modeling (SEM). An a priori sample size calculator for SEM was used for this purpose [25]. Considering the structural complexity of the model with four observed variables and one latent variable, an anticipated effect size of 0.10, a significance level of 0.05, and a desired statistical power of 0.80, the calculator indicated a minimum recommended sample size of *n* = 100.

Data was collected from 18 November 2024 to 19 February 2025 using an online survey distributed through a private Facebook group, “Autistic. Everything about autism”. Eligible participants were parents of a child diagnosed with autism spectrum disorder (ASD). The justification for joining the group was based on our scientific purpose. No additional sampling methods were applied.

Participants were informed about the study’s aims, procedures, and data usage. Confidentiality was maintained, as the survey was anonymous and did not collect any personally identifying information. They were also told that their responses would be used exclusively for scientific research.

A total of 404 mothers responded to our invitation to complete the survey, of which 5 were excluded because the autistic individuals of interest were older than 18 years. Therefore, 399 mothers’ reports about their autistic children were included in the analysis, representing both children and adolescents of different ages.

### 2.2. Questionnaire and Measures

Data was collected using an electronic self-administered questionnaire, which consisted of standardized scales and original questions designed in order to collect socio-demographic information on respondents’ sex, age, place of residence (urban or rural area), marital status, education, and number of children in the household. Child-related socio-demographic data included sex and age only. Parents were also asked to assess their child’s dental health status by rating it as 1—good; 2—satisfactory; or 3—bad. The severity of a child’s ASD was assessed through parental reporting; parents were asked in the questionnaire to indicate the autism severity level that had been previously assessed for their child by medical professionals according to the Childhood Autism Rating Scale (CARS) [26], categorized as 1—mild, 2—moderate, or 3—severe. To assess dietary habits, the Healthy Diet Index (HDI) was used [27], which evaluates the consumption frequency of 16 different food groups. The theoretical sums range from 16 (i.e., not reaching any “optimal” consumption frequency) to 80 (the maximum score). Higher values of the index indicate the highest odds of fulfilling a healthy dietary pattern. Based on the HDI score terciles, the authors of the index recommend classifying diet quality into three categories: 1—good; 2—satisfactory; or 3—poor [27]. Finally, parents were asked how their child typically reacts to oral hygiene procedures (e.g., toothbrushing willingness).

### 2.3. Data Analysis

First, descriptive statistics were calculated to estimate the frequency (*n*), percentage (%), mean, standard deviation (SD), and range of the variables. Spearman’s correlation ρ was calculated to evaluate the bivariate association between categorical ordinal variables. The chi-square (χ^2^) test and Bonferroni z-test were applied to compare percentages of categorical variables. The relationship between variables was illustrated using the Odds Ratio (OR). A *p*-value < 0.05 was considered statistically significant, and the confidence interval (CI) was set at 95%. These analyses were conducted using SPSS statistical software (version 21; IBM SPSS Inc., Chicago, IL, USA).

Next, a path model with direct and indirect effects was analyzed using Structural Equation Modeling (SEM) [23,24] and employed using MPLUS 8.11 [28]. Because each variable was represented by an ordered categorical (ordinal) variable, the associations between variables were estimated using probit regression, which is suitable for analyzing non-continuous variables [28,29,30]. The model produced the probit regression coefficients B, as well as standardized coefficients β (stdyx mode). The coefficients indicate how the likelihood of moving to a higher outcome level changes when the predictor increases by one unit while keeping other predictors constant. In this analysis, it was preferable to use the unstandardized regression weight B because, as in logistic regression, it is directly related to the Odds Ratio (OR) through the following equation: OR = exp (1.8 × B) [29]. Model parameters were estimated using the Weighted Least Squares Mean and Variance Adjusted (WLSMV) estimator, which is specifically designed for categorical observed variables and is robust to violations of normality [28,30]. Therefore, the use of probit regression with WLSMV estimation provided conceptually coherent latent-variable modeling for ordinal data and allowed stable estimation of direct and indirect effects in SEM. Such an approach is the dominant methodological choice for categorical structural models, whereas ordered logistic alternatives are less compatible with complex mediation pathways and yield less interpretable parameters in SEM contexts [28,30]. SEM analysis with the WLSMV estimator provided the following model fit indices to evaluate how well the model represents the data: Comparative Fit Index (CFI), Tucker–Lewis Index (TLI), Root Mean Square Error of Approximation (RMSEA), and Root Mean Square Error (RMSE) [23,30,31]. Smaller values (below 0.08) of RMSEA and RMSE indicated the acceptability of the model, while CFI and TLI values closer to 1 (≥0.90) showed a better fit of the model [23]. The χ^2^/(degree of freedom, df) statistic was used to assess the magnitude of the discrepancy between the sample and the fitted covariance matrix; its value being less than 3 or a non-significant *p*-value (*p* > 0.05) corresponds to an acceptable fit.

Finally, we examined whether regression weights and other path model estimates differed between boys and girls, as well as across age groups, using a two-step multigroup analysis approach [28]. In the first step, we estimated the structural model separately for each group. In the second step, we tested whether these associations differed significantly across groups by constraining all regression paths to be equal. The chi-square difference test (DIFFTEST) was then used to compare the constrained and unconstrained models. A *p*-value greater than 0.05 indicated no significant loss of fit, suggesting that the associations among autism, dietary quality, brushing behavior, and dental health were statistically equivalent across groups.

## 3. Results

### 3.1. Sample Characteristics

All questionnaires were completed exclusively by mothers, whose age ranged from 23 to 59 years, with a mean age of 38.1 years (SD = 6.4). Socio-demographic data (Table 1) indicates that respondents were likely to have a college or university education and that most were married. All families had a child with ASD, and in nearly one-third of the families (28.3%), the autistic child was the only child. The majority of the children (84.5%) were male. The children’s ages ranged from 2 to 18 years, with a mean age of 7.8 years (SD = 3.6). Based on the median age of 7, the children were divided into two groups approximately equal in size: younger (2–7 years, 52.6%) and older (8–18 years, 47.4%).

Across the sample, the dental health of 27.4% of children was reported to be good, 54.6% satisfactory, and 18.0% poor. According to the severity scale for autism spectrum disorder, the sample consisted primarily of children with moderate (40.4%) and severe (40.6%) levels of the condition. Based on the mothers’ responses to the HDI scale questions, the diet of most children (72.4%) was found to be of poor quality. Finally, the questionnaire revealed varying levels of willingness to brush teeth, with more than half (51.4%) of the autistic children showing complete unwillingness or strong reluctance to brush their teeth. It was observed that this behavior was more common among younger children (2–7 years old) than older children (56.7% and 45.5%, respectively; *p* < 0.05 according to the Bonferroni test).

### 3.2. Bivariate Relationships

Bivariate relationships among the severity of autism spectrum disorder (ASD), dental health status, diet quality, and toothbrushing willingness are presented in Supplementary Table S1. These relationships are also illustrated graphically.

**Figure 1:**
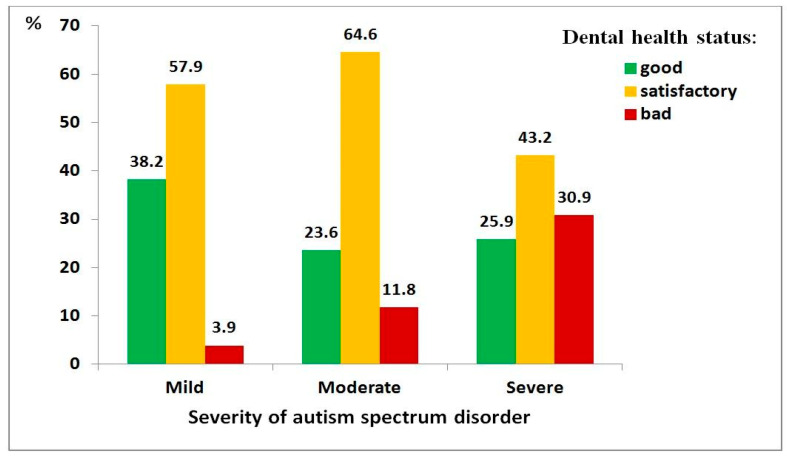
Distribution of dental health status among children with autism spectrum disorder, by severity level: mild (*n* = 76), moderate (*n* = 161), severe (*n* = 162).

**Figure 2:**
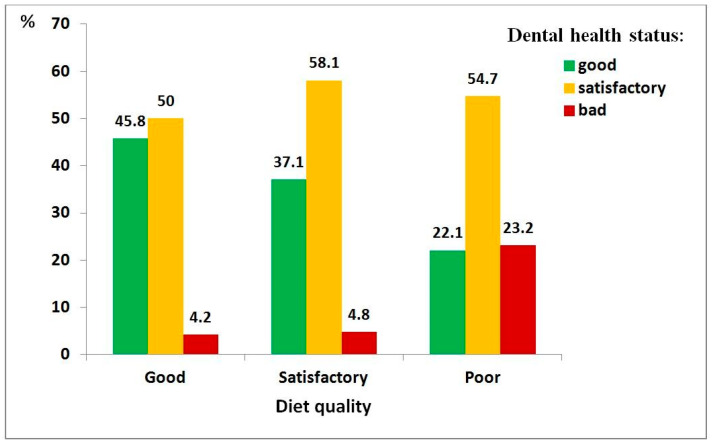
Distribution of dental health status among children with autism spectrum disorder, by diet quality: good (*n* = 48), satisfactory (*n* = 62), poor (*n* = 280).

**Figure 3:**
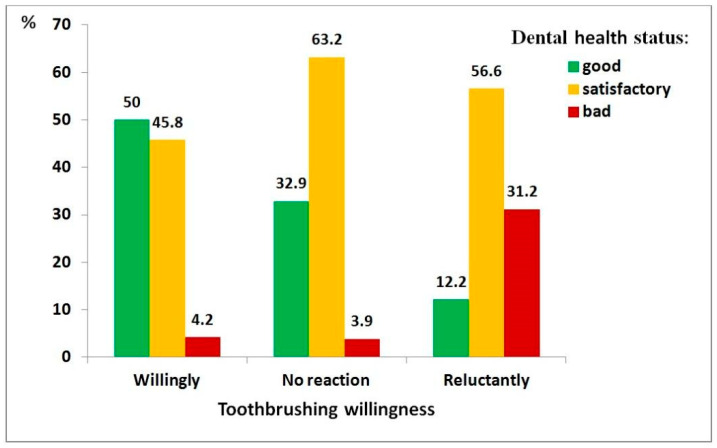
Distribution of dental health status among children with autism spectrum disorder, by toothbrushing willingness: willingly (*n* = 118), no reaction (*n* = 76), reluctantly (*n* = 205).

Figure 1, Figure 2 and Figure 3 present the relationships between dental health status and three key factors: ASD severity, diet quality, and toothbrushing willingness. It was considered that these factors had a direct effect on dental health status. In each case, a statistically significant relationship was observed, with poorer outcomes linked to poorer dental health.

Regarding ASD severity (Figure 1), greater disorder severity was associated with worse dental health (χ^2^ = 37.74, df = 4, *p* < 0.001; ρ = 0.213, *p* < 0.001). Children with severe ASD were substantially more likely to have poor dental health compared to those with mild ASD (OR = 12.7, 95% CI: 3.6–44.4).

Regarding diet quality (Figure 2), children with a poor-quality diet showed significantly higher odds of poor dental health (χ^2^ = 26.69, df = 4, *p* < 0.001; ρ = 0.246, *p* < 0.001), with an 11-fold increase in risk compared to those with a good-quality diet (95% CI: 2.5–48.6).

Regarding toothbrushing willingness (Figure 3), the association was particularly strong (χ^2^ = 83.66, df = 4, *p* < 0.001; ρ = 0.435, *p* < 0.001). Children unwilling to brush their teeth had 30 times higher odds of poor dental health than those who were willing (95% CI: 10.8–83.2).

Diet quality and toothbrushing willingness play a mediating role in the relationship between ASD and dental health; therefore, it was important to assess each of their relationships with ASD severity.

**Figure 4:**
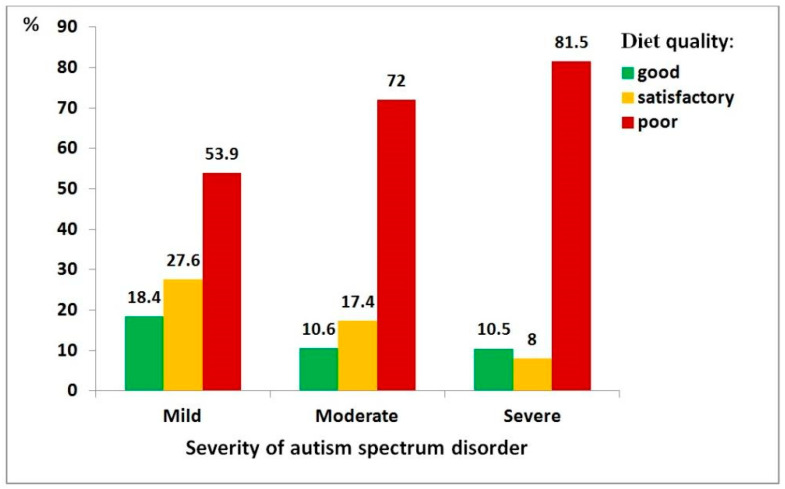
Distribution of diet quality among children with autism spectrum disorder, by severity level: mild (*n* = 76), moderate (*n* = 161), severe (*n* = 162).

Figure 4 shows the relationship between ASD severity and diet quality, demonstrating that greater disorder severity is associated with poorer diet quality—an effect that was statistically significant (χ^2^ = 22.00, df = 4, *p* < 0.001; ρ = 0.176, *p* < 0.001). It can be illustrated with the following example: children with severe ASD had a significantly higher risk of poor diet quality compared to those with mild ASD (OR = 2.53, 95% CI: 1.15–5.55).

**Figure 5:**
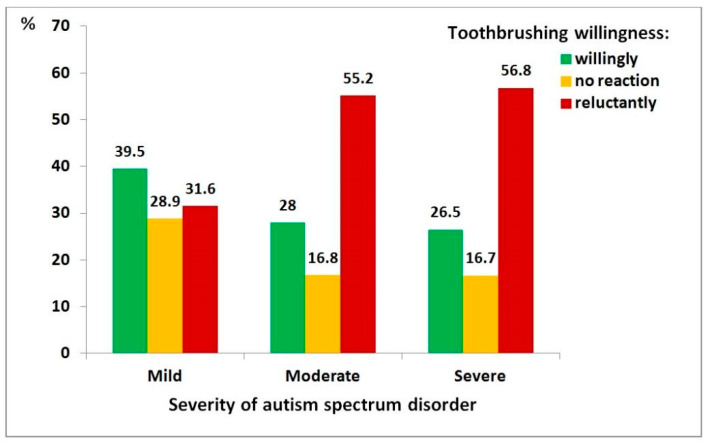
Distribution of toothbrushing willingness among children with autism spectrum disorder, by severity level: mild (*n* = 76), moderate (*n* = 161), severe (*n* = 162).

Figure 5 presents the relationship between ASD severity and children’s willingness to brush their teeth. This association was also statistically significant (χ^2^ = 15, df = 4, *p* = 0.004), though the correlation between variables was relatively weak (ρ = 0.140, *p* = 0.005). Children with severe ASD were nearly three times more likely to be reluctant to brush their teeth compared to those with mild ASD (OR = 2.85, 95% CI: 1.50–5.42).

### 3.3. MPLUS Path Model

**Figure 6:**
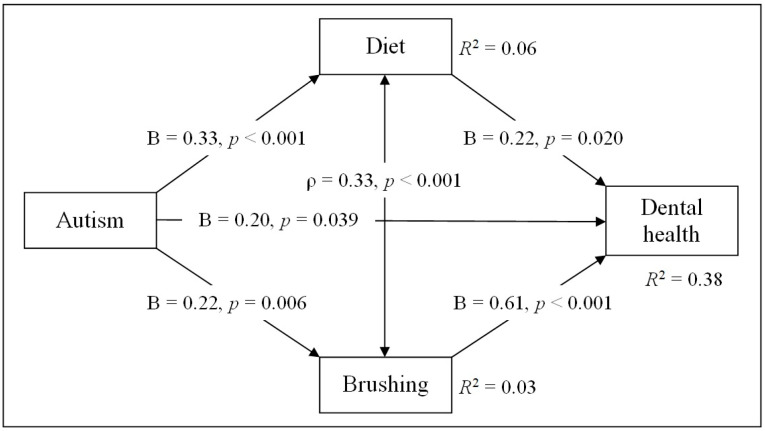
Path diagram of the association between autism and dental health. Meaning and codes of variables: Autism—severity of autism spectrum disorders: 1—mild; 2—moderate; 3—severe. Dental health—dental health status: 1—good; 2—satisfactory; 3—bad. Diet—diet quality: 1—good; 2—satisfactory; 3—poor. Brushing—toothbrushing willingness: 1—willingly; 2—no reaction; 3—reluctantly. Parameters: B = unstandardized probit coefficient; ρ = correlation coefficient; R^2^ = explained variance in each dependent variable.

Initially, the path model included a direct effect of autism spectrum disorder (ASD) severity on dental health status (B = 0.125, *p* = 0.262), as well as indirect effects through children’s diet quality (B = 0.325, *p* < 0.001, followed by B = 0.450, *p* < 0.001) and toothbrushing willingness (B = 0.223, *p* = 0.006, followed by B = 0.735, *p* < 0.001). However, this version of the model demonstrated an inadequate fit (CFI = 0.887 (not ≥0.90), TLI = 0.320 (not ≥0.90), RMSEA = 0.237 (not below 0.08), SRMR = 0.075). The modification index suggested adding an interaction between diet quality and toothbrushing willingness, as these variables were significantly correlated (ρ = 0.331, *p* < 0.001). After including this interaction, the model became saturated (df = 0) and achieved a perfect fit (CFI = 1.000, TLI = 1.000, RMSEA = 0.000, SRMR = 0.000) (Table 2). The path diagram of the final model is presented in Figure 6.

The path analysis indicated that autistic children tend to have poorer dental health. The effect of ASD severity on dental health status was small to moderate (B = 0.199, *p* = 0.039). Better diet quality was significantly associated with improved dental health, while children’s toothbrushing willingness had the strongest positive effect on dental health. In addition, children with more severe ASD symptoms tended to choose a less healthy diet and exhibit less optimal toothbrushing behavior. The model accounted for a meaningful proportion (about 38%) of the variance in dental health (R^2^ = 0.375). By contrast, ASD severity accounted for only about 6% of the variance in diet quality and 3% in toothbrushing willingness, indicating that most of the variation in these behaviors remains unexplained.

The sensitivity analyses showed that the overall pattern of direct and indirect effects in this path model remained consistent across alternative variable categorizations, age- and sex-specific subsamples, and model variations with or without the brushing–diet correlation.

### 3.4. Direct and Indirect Effects

From the regression of dental health status on ASD severity, the direct effect, as mentioned above, was as follows: unstandardized B = 0.199 (*p* = 0.039), standardized (std) β = 0.117. According to the path model, autism influences dental health through two paths: diet and toothbrushing behaviors. Then, for the first path, the indirect effect = 0.325 × 0.216 ≈ 0.070 (std: 0.235 × 0.175 ≈ 0.041), and for the second path, the indirect effect = 0.223 × 0.613 ≈ 0.137 (std: 0.174 × 0.490 ≈ 0.080). The total indirect effect is as follows: unstandardized 0.070 + 0.137 ≈ 0.207, std 0.041 + 0.080 ≈ 0.121. Overall, autism has a significant total (direct and indirect) effect on dental health (0.406; std: 0.238).

The presented estimations show that the total indirect effect of ASD on dental health (0.207, *p* = 0.026) was approximately as strong as the direct effect (0.199, *p* = 0.039). This means that about half of autism’s total impact on dental health is explained indirectly through brushing habits and eating behavior. The indirect effect via toothbrushing willingness (0.137; std 0.080) is more than twice as large as the indirect effect via diet choice (0.070; std 0.041). Thus, brushing behavior is the stronger mediator linking autism to dental health.

### 3.5. Multigroup Comparison

This analysis allows us to test whether structural paths differ across groups, namely between boys and girls and across children’s age groups.

#### 3.5.1. Boys vs. Girls

In the unconstrained multigroup model, the structural associations were estimated separately for boys and girls. Among boys, ASD severity showed significant associations with poorer diet (B = 0.347, *p* < 0.001) and lower toothbrushing willingness (B = 0.241, *p* = 0.007). Both behaviors were subsequently linked to poorer dental health (diet → dental health: B = 0.212, *p* = 0.044; brushing → dental health: B = 0.669, *p* < 0.001). Autism also showed a borderline direct effect on dental health (B = 0.204, *p* = 0.054). In contrast, the corresponding paths among girls were weaker and largely non-significant, likely reflecting the smaller sample size (Table 3, Step 1).

To test whether these associations differed by children’s sex, all structural paths were constrained to equality across groups (Table 3, Step 2). The constrained model fit the data well (χ^2^/df = 1.25, *p* = 0.275; RMSEA = 0.034; CFI = 0.993; TLI = 0.986; SRMR = 0.045). The chi-square difference test indicated no significant loss of fit relative to the unconstrained model (DIFFTEST, *p* = 0.275). This indicates that the structural relations between autism, diet, brushing, and dental health can be considered statistically equivalent for boys and girls. In the final constrained model, toothbrushing willingness emerged as the strongest predictor of dental health for both sexes (B = 0.614, *p* < 0.001), followed by smaller but significant effects of diet (B = 0.209, *p* = 0.021) and autism severity (B = 0.208, *p* = 0.033).

#### 3.5.2. Younger vs. Older Subjects

We next examined whether the structural path differed between younger (2–7 years) and older (8–18 years) children (Table 4).

In the unconstrained model, estimated separately for the two age groups, toothbrushing willingness strongly predicted dental health in both age groups, whereas the direct effect of ASD severity on dental health was not significant. ASD severity was significantly associated with dietary quality among younger children (B = 0.407, *p* < 0.001), while among older children it was more strongly related to toothbrushing behavior (B = 0.317, *p* = 0.006). These patterns suggest possible age-related differences in the pathways linking ASD severity to oral health behaviors.

In the second step, all structural paths were constrained to equality. The constrained model showed an excellent fit (χ^2^/df = 0.54, *p* = 0.777; RMSEA = 0.00; CFI = 1.00; SRMR = 0.038), and the chi-square difference test indicated no significant loss of fit compared with the unconstrained model. Thus, despite some descriptive differences between age groups, the associations among autism severity, diet, brushing behavior, and dental health can be considered statistically equivalent across younger and older children.

## 4. Discussion

This study revealed both significant direct and indirect effects of parent-reported ASD severity on dental health outcomes (also parent-reported) among individuals aged 2 to 18 years. The indirect effects, particularly those mediated through toothbrushing willingness and dietary choices, contributed almost as much as the direct effects. Across both sexes and age groups, toothbrushing behavior emerged as the strongest mediator, while eating behavior played a smaller but still meaningful role.

Evidence indicates that the relationship between ASD and dental health is complex and influenced by multiple factors, including behavioral difficulties such as low toothbrushing willingness and poor dietary choices [32,33]. Analyzing such multifaceted associations requires modern statistical tools. For this reason, we applied Structural Equation Modeling (SEM), which is well-suited for examining direct and indirect pathways between health-related variables and health outcomes. To our knowledge, no peer-reviewed studies have used SEM to model the pathways from ASD severity through mediating behaviors (e.g., toothbrushing, diet) to dental health outcomes. This may be because most ASD–oral health studies are descriptive, case–control, or cross-sectional and rely on simpler statistical techniques (e.g., *t*-tests, chi-square tests, logistic regression) rather than advanced modeling approaches. For example, recent studies commonly report caries prevalence, plaque index, and oral health behaviors but do not employ SEM [32,33]. Therefore, our study contributes a novel and methodologically advanced perspective, offering deeper insights into dental health etiology in autistic children and adolescents.

When interpreting the study results, it is important to consider the specific traits of individuals with ASD. Some of the identified patterns may also occur in typically developing children and adolescents. For instance, there is evidence that poor oral hygiene is a risk factor for dental health in children [34]. The state of oral hygiene is most often associated with how often the child brushes their teeth with a toothbrush and toothpaste. However, this study explored whether the child brushes their teeth willingly. The answers to this question reflect the child’s attitudes, which, as stated by Ajzen’s Theory of Planned Behavior [35], determine the intention to act in a certain way, in this specific case, to brush teeth. It has been observed that autistic individuals demonstrate attitudes that are difficult to change, and these correlate with ASD severity [36]; therefore, our chosen question, intended to assess oral hygiene status, was logical.

On the other hand, individuals suffering from autism disorders often have increased or decreased sensory sensitivity, which can make it difficult to tolerate the sensation of a toothbrush, the taste of toothpaste, or dental procedures. The consequence of this poorer oral hygiene is increased plaque and a higher risk of dental caries. Therefore, the pathway of autism’s impact on dental health status through toothbrushing is logical and significant [13].

The study determined that another pathway of autism’s impact on dental health manifests in dietary choices. There is abundant evidence that unbalanced nutrition is negatively associated with dental health [37,38,39]. In addition, autistic individuals often have specific dietary preferences [14,15,40]. Autism is characterized by selective eating. This means individuals choose a limited range of food products, with a preference for softer, easier-to-chew, or sweeter foods, which can increase the risk for dental caries. Furthermore, not eating certain foods may lead to deficiencies in vitamins or minerals (e.g., calcium, vitamin D), which can affect the condition of dental bone tissue [37]. The indirect impact of autism on dental health, which may be mediated by dietary choice, has also been confirmed by other authors [14,40]. Thus, the mechanism of autism’s indirect effect on dental health is easily explained.

The path model explored in the present study also suggested a significant direct effect of autism on dental health, approximately as strong as the total indirect effect through brushing habits and eating behavior. This “direct effect” should not be interpreted as a biological impact of ASD itself but rather as variance likely related to behavioral or systemic factors. Recent research indicates that ASD may have a direct impact on oral health through certain mechanisms. Some autistic people exhibit self-harming oral habits such as bruxism, object chewing, and teeth clenching, which can have a direct adverse effect on their dental health [41,42,43]. In addition, there is a hypothesis about the side effects of medications prescribed for the treatment of comorbid conditions of autism influencing oral health [44]. For example, certain psychoactive medications may reduce saliva flow, which can contribute to caries formation [45]. Furthermore, individuals with ASD tend to keep food in their mouths for prolonged periods of time, which can also increase the risk for dental caries [46]. Finally, many individuals with ASD experience challenges with manual dexterity and muscle tone, which can make effective toothbrushing difficult to achieve and lead to insufficient oral hygiene [46]. Therefore, the direct pathway seen in our model is empirically supported by these results.

The relationships described in the path model were also compared between sex groups of children and adolescents. In this case, no significant group difference was found. Even though the absolute difference in some estimates between boys and girls (e.g., the correlation between diet quality and toothbrushing willingness) was large, it did not reach significance. This was likely constrained by the small sample size of the girls’ group.

The study included participants with a wide age range (from 2 to 18 years). It is evident that such individuals’ attitudes and behaviors may have differed depending on their age [47,48,49]. However, the study revealed that the relationships described in the path model were equally characteristic of both younger and older autistic individuals, although the descriptive results suggested that autism was more strongly related to dietary quality in younger participants and to brushing in older participants. This finding implies that the pathways linking autism with oral health behaviors and outcomes are largely consistent across the lifespan. Brushing habits emerged as the most robust and consistent predictor of dental health, demonstrating the importance of promoting regular oral hygiene practices in both younger and older individuals with ASD. At the same time, the observed trends point to the support of healthier dietary choices in younger populations, providing additional benefits [38,39,50].

The equivalence of these associations across sex and age groups suggests that tailored interventions targeting oral hygiene behaviors and nutrition may be broadly applicable across diverse subgroups of autistic youth. In addition, creating comprehensive multidisciplinary care strategies requires an understanding of the direct and indirect effects of autism spectrum disorder on dental health. More accessible targeted interventions of different specialists that cover behavioral therapies, dietary habits, and dental care services are necessary to address these vulnerabilities. Children and adolescents with autism can benefit from improved oral health outcomes through partnerships between educators, caregivers, and healthcare professionals, which would greatly improve their general health. According to the results of our study, when designing preventive oral health programs involving an interdisciplinary team, it is recommended to differentiate patients according to the severity of their ASD. Both oral hygiene and diet management, as well as various visual and pedagogical tools, will be ineffective in severe cases of ASD, when even daily oral hygiene practices can be very difficult to implement. In such cases, general anesthesia may be justified even for diagnostic dental examination alone, but only for severe ASD, when all other methods are not effective [51].

Finally, particular attention should be paid to clarifying the definitions of “direct” and “indirect” effects, which were the focus of this study. We selected these terms based on an analysis of the available data using a path model that links dental health in autistic children and adolescents with ASD severity, toothbrushing behavior, and dietary choices (see Figure 6). However, alternative definitions of the terms “direct” and “indirect” effects can be found in the literature. A scoping review by Jones et al. [52] provided a systematic identification of barriers as many “indirect” factors (access, clinician education, and negative experiences), alongside “direct” sensory/behavioral issues. A systematic review by Erwin et al. [20] analyzed many studies reporting both direct behavioral/oral hygiene challenges and indirect external/contextual constraints (caregiver knowledge/attitudes and system-level accessibility). A narrative literature review by Angelova et al. [53] focused on clinical risks, behavioral barriers, the role of caregivers, and interventions. Therefore, it helps us separate what is directly associated with the child (sensory and behavioral factors) from what is mediated (care practices and systemic support). According to the reviews provided above, the classification of factors into “direct” and “indirect” was based on the proximity and nature of their impact on oral health. Thus, “direct” factors are those that stem directly from the core characteristics of autism (sensory, behavioral, communication, or motor differences). They influence oral health without requiring external mediators. In contrast, “indirect” factors are influences that arise as secondary or contextual consequences of autism. They are related to oral health through intermediate pathways, such as medication side effects, caregiver dynamics, or healthcare access. Therefore, given the differences in how the terms “direct” and “indirect” effects are conceptually defined, the results of this study should be interpreted with caution.

In addition to the previously mentioned limitation, this study has several other limitations worth mentioning. First, the study utilized SEM methods to analyze cross-sectional data. As a result, the sequence of events and temporal changes could not be identified. Since the development of oral diseases is chronic and progressive, longitudinal studies are essential for understanding the evolving relationship between ASD and oral health over time, as well as for establishing causality. Second, the high male predominance in the study sample (84.5%), which is consistent with male–female ratio of ASD prevalence in Europe (4.2 to 1) [54], may reduce the external validity of our study. Therefore, results should be interpreted with caution, particularly regarding autistic girls, who may present different behavioral or dental health profiles. Furthermore, our study relied only on respondents’ self-reported data from an online survey shared via Facebook and was thus subject to potential response and selection bias. For example, the responses to the questions about toothbrushing willingness and dental status were subjective and could be affected by the mother’s level of attachment to her child. It is important to mention that parental reports may be influenced by ASD severity itself. Mothers of children with more severe symptoms may perceive and report dental or behavioral problems differently from those of children with milder ASD. Such severity-related reporting tendencies may contribute to measurement error. In addition, even though evaluation of dental health using a 3-level ordinal scale ensured that parents with varying educational backgrounds could complete the survey consistently, when clinical examinations are not feasible, it is subject to information and misclassification bias. Another significant limitation related to parent-reported information on their child’s dental health status is that it could partially contribute to differences between the observed findings and clinical prevalence patterns. In addition, the lack of clinical validation of dental conditions should be considered, as the absence of confirmatory dental assessments may have affected the precision of the reported dental outcomes. Finally, because ASD diagnosis was parent-reported and severity was estimated using the Childhood Autism Rating Scale (CARS), some degree of diagnostic misclassification is possible. Therefore, future studies in this field should include clinical examinations of dental hygiene and oral health status. Furthermore, recruited participants from a specific online parents group could be more motivated, better informed or more proactive regarding their children’s dental health than the general population of caregivers of autistic children. This has clear implications for external validity, as the findings may overestimate positive behaviors or awareness of dental issues and may underestimate barriers experienced by families with lower engagement, limited access to online communities or different socioeconomic circumstances. Therefore, future studies should consider more diverse recruitment approaches to mitigate this bias. Moreover, because we used the Healthy Diet Index (HDI) to assess diet quality, we did not inquire about specific food consumption or preferences, which could be explored in future studies. Furthermore, our study was limited to analyzing only three factors for dental health. Although these factors explained a significant portion (37%) of the variance in dental health status, the inclusion of more factors like medication use, comorbidities, sensory sensitivities, socioeconomic background, and access to dental services may provide a broader view of multifactorial dental disease in autistic children [15,55,56]. Finally, our study was also limited because the social factors of the respondents (age, education, etc.) were not included in the analysis. Omission of these factors, mentioned above, means that we cannot exclude the possibility that some of the associations observed in our study are partially or fully explained by these unmeasured variables. Because our data collection tool did not include detailed information on these confounders, it limits the interpretability of our findings. Future longitudinal and clinically based studies are needed to overcome the listed limitations.

Despite the limitations mentioned, the results of this study offer some insights into the relationship between ASD and dental health. These findings may also be valuable in developing intervention strategies for families with autistic children and adolescents.

## 5. Conclusions

This study shows that dental health in autistic children and adolescents is influenced by both ASD severity and everyday oral health behaviors, as reported by their parents. While ASD severity is associated with poorer dental outcomes, toothbrushing willingness and dietary quality represent the key modifiable factors linked to dental health. These pathways were consistent across sex and age groups of younger and older children, suggesting that core preventive strategies may be broadly applicable across the autistic population. Interventions that support regular toothbrushing and healthier dietary habits may offer the greatest benefit. Integrating behavioral, nutritional, and dental guidance into family-centered care could help reduce dental health disparities among autistic youth.

## Figures and Tables

**Table 1:** Socio-demographic and clinical characteristics of the sample (*n* = 399).

Characteristic	*n*	%	Mean (SD)
Age of respondents (mothers):			38.1 (6.4)
23–39 years	244	61.2	
40–59 years	155	38.8	
Mother’s education:			
Basic/secondary	133	33.3	
College/university	266	66.7	
Marital status:			
Married	332	83.2	
Single	67	16.8	
Number of children in family:			
One	113	28.3	
Two	183	45.9	
Three or more	103	25.8	
Household location:			
Urban area	341	85.5	
Rural area	58	14.5	
Sex of children:			
Boys	337	84.5	
Girls	62	15.5	
Age of children:			7.8 (3.6)
2–7 years	210	52.6	
8–18 years	189	47.4	
Dental health status:			
Good	109	27.4	
Satisfactory	218	54.6	
Bad	72	18.0	
Severity of autism spectrum disorders:			
Mild	76	19.0	
Moderate	161	40.4	
Severe	162	40.6	
Diet:			
Good	48	12.0	
Satisfactory	62	15.5	
Poor	289	72.4	
Toothbrushing willingness:			
Willingly	118	29.6	
No reaction	76	19.0	
Reluctantly	205	51.4	

**Table 2:** Path coefficients from Mplus model (WLSMV estimation, *n* = 399).

Path	B	SE	z	*p*-Value	β	R^2^ (*p*-Value)
Dependent Variable: Dental health						0.375 (*p* < 0.001)
Autism → Dental health	0.199	0.096	2.06	0.039	0.117	
Diet → Dental health	0.216	0.093	2.33	0.020	0.175	
Brushing → Dental health	0.613	0.088	6.99	<0.001	0.491	
Dependent Variable: Diet						0.055 (*p* = 0.048)
Autism → Diet	0.325	0.087	3.76	<0.001	0.235	
Dependent Variable: Brushing						0.027 (*p* = 0.158)
Autism → Brushing	0.223	0.081	2.76	0.006	0.163	
Correlation						
Brushing ↔ Diet	0.331	0.068	4.85	<0.001	0.331	

Notes: B = unstandardized probit coefficient; SE = standard error; z = B/SE; β = standardized coefficient; R^2^ = explained variance in each dependent variable.

**Table 3:** Comparison of path coefficients between boys and girls.

Path	Boys (*n* = 337)			Girls (*n* = 62)		
	B	*p*-Value	R^2^ (*p*-Value)	B	*p*-Value	R^2^ (*p*-Value)
*Step 1. Unconstrained model*						
Dependent Variable: Dental health			0.413 (<0.001)			0.202 (0.119)
Autism → Dental health	0.204	0.054		0.169	0.548	
Diet → Dental health	0.212	0.044		0.209	0.243	
Brushing → Dental health	0.669	<0.001		0.394	0.024	
Dependent Variable: Diet			0.061 (0.059)			0.038 (0.509)
Autism → Diet	0.347	<0.001		0.247	0.201	
Dependent Variable: Brushing			0.022 (0.165)			0.017 (0.660)
Autism → Brushing	0.241	0.007		0.163	0.386	
Correlation						
Brushing ↔ Diet	0.383	<0.001		0.010	0.959	
*Step 2. Constrained model*						
Dependent Variable: Dental health			0.381 (<0.001)			0.389 (<0.001)
Autism → Dental health	0.208	0.033		0.208	0.033	
Diet → Dental health	0.209	0.021		0.209	0.021	
Brushing → Dental health	0.624	<0.001		0.624	<0.001	
Dependent Variable: Diet			0.054 (0.048)			0.065 (0.056)
Autism → Diet	0.327	<0.001		0.327	<0.001	
Dependent Variable: Brushing			0.027 (0.151)			0.032 (0.158)
Autism → Brushing	0.227	0.005		0.227	0.005	
Correlation						
Brushing ↔ Diet	0.335	<0.001		0.335	<0.001	

Notes: B = unstandardized coefficient; R^2^ = explained variance in each dependent variable.

**Table 4:** Comparison of path coefficients between children’s age groups.

Path	2–7-Year-Olds (*n* = 210)			8–23-Year-Olds (*n* = 194)		
	B	*p*-Value	R^2^ (*p*-Value)	B	*p*-Value	R^2^ (*p*-Value)
*Step 1. Unconstrained model*						
Dependent Variable: Dental health			0.390 (<0.001)			0.377 (<0.001)
Autism → Dental health	0.212	0.128		0.215	0.115	
Diet → Dental health	0.152	0.230		0.292	0.033	
Brushing → Dental health	0.685	<0.001		0.548	<0.001	
Dependent Variable: Diet			0.082 (0.068)			0.033 (0.319)
Autism → Diet	0.407	<0.001		0.245	0.053	
Dependent Variable: Brushing			0.011 (0.537)			0.054 (0.153)
Autism → Brushing	0.141	0.221		0.314	0.006	
Correlation						
Brushing ↔ Diet	0.357	<0.001		0.309	0.001	
*Step 2. Constrained model*						
Dependent Variable: Dental health			0.380 (<0.001)			0.382 (<0.001)
Autism → Dental health	0.198	0.041		0.198	0.041	
Diet → Dental health	0.224	0.016		0.224	0.016	
Brushing → Dental health	0.616	<0.001		0.616	<0.001	
Dependent Variable: Diet			0.056 (0.048)			0.058 (0.047)
Autism → Diet	0.331	<0.001		0.331	<0.001	
Dependent Variable: Brushing			0.027 (0.155)			0.029 (0.155)
Autism → Brushing	0.228	0.005		0.228	0.005	
Correlation						
Brushing ↔ Diet	0.334	<0.001		0.334	<0.001	

Notes: B = unstandardized coefficient; R^2^ = explained variance in each dependent variable.

## Data Availability

The data presented in this study are available on request from the corresponding author.

## Supplementary Materials

The following supporting information can be downloaded at https://www.mdpi.com/article/10.3390/medicina62010086/s1, Table S1: Bivariate relationships between main study variables: severity of autism spectrum disorder (ASD), dental health status, diet quality, and toothbrushing willingness.
