# Supplementary material for: Direct and Indirect Effects of Autism Spectrum Disorder Severity on Dental Health Status in Children and Adolescents: A Structural Equation Modeling Approach
Source: Medicina (Kaunas). 2025 Dec 31;62(1):86. doi: 10.3390/medicina62010086 (PMC12843695; doi:10.3390/medicina62010086)
Supplement: Supplementary file 1 [file medicina-62-00086-s001.zip › medicina-4001833-supplementary.pdf]

Table S1. Bivariate relationships between main study variables: severity of autism spectrum disorder (ASD), dental health status, diet quality, and toothbrushing willingness.

Severity of ASD	Dental health status							
	Good		Satisfactory		Bad		Total	
	n	(%)	*n*	(%)	*n*	(%)	*n*	(%)
Mild	29	(38.2)	44	(57.9)	3	(3.9)	76	(100)
Moderate	38	(23.6)	104	(64.6)	19	(11.8)	161	(100)
Severe	42	(25.9)	70	(43.2)	50	(30.9)	162	(100)
Total	109	(27.3)	218	(54.6)	72	(18.0)	399	(100)
Assessments of the relationship between variables: $\chi^2 = 37.74$, df = 4, $p < 0.001$; and $\rho = 0.213$, $p < 0.001$ (See Figure 1)								

Diet quality	Dental health status							
	Good		Satisfactory		Bad		Total	
	n	(%)	*n*	(%)	*n*	(%)	*n*	(%)
Good	22	(45.8)	24	(50.0)	2	(4.2)	48	(100)
Satisfactory	23	(37.1)	36	(58.1)	3	(4.8)	62	(100)
Poor	64	(22.1)	158	(54.7)	67	(23.2)	289	(100)
Total	109	(27.3)	218	(54.6)	72	(18.0)	399	(100)
Assessments of the relationship between variables: $\chi^2 = 26.69$, df = 4, $p < 0.001$; and $\rho = 0.246$, $p < 0.001$ (See Figure 2)								

Toothbrushing willingness	Dental health status							
	Good		Satisfactory		Bad		Total	
	n	(%)	*n*	(%)	*n*	(%)	*n*	(%)
Willingly	59	(50.0)	54	(45.8)	5	(4.2)	118	(100)
No reaction	25	(32.9)	48	(63.2)	3	(3.9)	76	(100)
Reluctantly	25	(12.2)	116	(56.6)	64	(31.2)	205	(100)
Total	109	(27.3)	218	(54.6)	72	(18.0)	399	(100)
Assessments of the relationship between variables: $\chi^2 = 83.66$, df = 4, $p < 0.001$; and $\rho = 0.435$, $p < 0.001$ (See Figure 3)								

Severity of ASD	Diet quality							
	Good		Satisfactory		Poor		Total	
	n	(%)	*n*	(%)	*n*	(%)	*n*	(%)
Mild	14	(18.4)	21	(27.6)	41	(53.9)	76	(100)
Moderate	17	(10.6)	28	(17.4)	116	(72.0)	161	(100)
Severe	17	(10.5)	13	(8.0)	132	(81.5)	162	(100)
Total	48	(12.0)	62	(15.5)	289	(72.4)	399	(100)
Assessments of the relationship between variables: $\chi^2 = 22.00$, df = 4, $p < 0.001$; and $\rho = 0.176$, $p < 0.001$ (See Figure 4)								

Severity of ASD	Toothbrushing willingness							
	Willingly		No reaction		Reluctantly		Total	
	n	(%)	*n*	(%)	*n*	(%)	*n*	(%)
Mild	30	(39.5)	22	(28.9)	24	(31.6)	76	(100)
Moderate	45	(28.0)	27	(16.8)	89	(55.3)	161	(100)
Severe	43	(26.5)	27	(16.7)	92	(56.8)	162	(100)
Total	118	(29.6)	76	(19.0)	205	(51.4)	399	(100)
Assessments of the relationship between variables: $\chi^2 = 15.20$, df = 4, $p = 0.004$; and $\rho = 0.140$, $p = 0.005$ (See Figure 5)								
